# Athletic Cardiac Adaptation in Males Is a Consequence of Elevated Myocyte Mass

**DOI:** 10.1161/CIRCIMAGING.115.003579

**Published:** 2016-04-19

**Authors:** Adam K. McDiarmid, Peter P. Swoboda, Bara Erhayiem, Rosalind E. Lancaster, Gemma K. Lyall, David A. Broadbent, Laura E. Dobson, Tarique A. Musa, David P. Ripley, Pankaj Garg, John P. Greenwood, Carrie Ferguson, Sven Plein

**Affiliations:** From the Multidisciplinary Cardiovascular Research Centre (MCRC) and Leeds Institute of Cardiovascular and Metabolic Medicine (A.K.M., P.P.S., B.E., D.A.B., L.E.D., T.A.M., D.P.R., P.G., J.P.G., S.P.), and Division of Biomedical Imaging, Multidisciplinary Cardiovascular Research Centre (MCRC) and School of Biomedical Sciences (R.E.L., G.K.L., C.F.), University of Leeds, Clarendon Way, Leeds, UK.

**Keywords:** athlete’s heart, cardiovascular magnetic resonance imaging, ECV, exercise physiology, hypertrophy/remodeling, T1 mapping

## Abstract

**Background—:**

Cardiac remodeling occurs in response to regular athletic training, and the degree of remodeling is associated with fitness. Understanding the myocardial structural changes in athlete’s heart is important to develop tools that differentiate athletic from cardiomyopathic change. We hypothesized that athletic left ventricular hypertrophy is a consequence of increased myocardial cellular rather than extracellular mass as measured by cardiovascular magnetic resonance.

**Methods and Results—:**

Forty-five males (30 athletes and 15 sedentary age-matched healthy controls) underwent comprehensive cardiovascular magnetic resonance studies, including native and postcontrast T1 mapping for extracellular volume calculation. In addition, the 30 athletes performed a maximal exercise test to assess aerobic capacity and anaerobic threshold. Participants were grouped by athleticism: untrained, low performance, and high performance (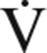
O_2max_ <60 or>60 mL/kg per min, respectively). In athletes, indexed cellular mass was greater in high- than low-performance athletes 60.7±7.5 versus 48.6±6.3 g/m^2^; *P*<0.001), whereas extracellular mass was constant (16.3±2.2 versus 15.3±2.2 g/m^2^; *P*=0.20). Indexed left ventricular end-diastolic volume and mass correlated with 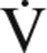
O_2max_ (*r*=0.45, *P*=0.01; *r*=0.55, *P*=0.002) and differed significantly by group (*P*=0.01; *P*<0.001, respectively). Extracellular volume had an inverse correlation with 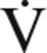
O_2max_ (*r*=−0.53, *P*=0.003 and left ventricular mass index (r=-0.44, *P*=0.02).

**Conclusions—:**

Increasing left ventricular mass in athlete’s heart occurs because of an expansion of the cellular compartment while the extracellular volume becomes relatively smaller: a difference which becomes more marked as left ventricular mass increases. Athletic remodeling, both on a macroscopic and cellular level, is associated with the degree of an individual’s fitness. Cardiovascular magnetic resonance ECV quantification may have a future role in differentiating athlete’s heart from change secondary to cardiomyopathy.

Regular exercise training places demands on the heart that may lead to cardiac remodeling. The rare but prominent cases of sudden cardiac death in elite athletes have underlined the importance of understanding the adaptation underlying athlete’s heart (AH).^1^ Gaining insights into the remodeling process is important to understand the nature of adaptation and to develop tools that distinguish AH from cardiomyopathic change, particularly those of hypertrophic cardiomyopathy (HCM).

**See Editorial by Graham-Brown and McCann**

**See Clinical Perspective**

Cardiovascular magnetic resonance (CMR) imaging is particularly well suited to investigate changes of structure and function in athletic cardiac remodeling because of its multiparametric capabilities, high spatial resolution, and lack of ionizing radiation. The excellent reproducibility of the method means that anatomic change can be quantified with greater confidence and smaller sample size than with other methods.^2,3^ The emerging CMR technique of extracellular volume (ECV) quantification allows the relative volumes of the extracellular and intracellular myocardial compartments to be quantified. This method has been used to quantify extracellular expansion (higher ECV) secondary to interstitial fibrosis in cardiomyopathy^4^ and conversely greater myocyte volume (lower ECV) in pathologies such as Anderson–Fabry disease.^5,6^

In this study, we sought to characterize cardiac adaptation in male athletes using CMR to gain insights into the mechanisms underlying athletic remodeling. We hypothesized that in athletes, left ventricular (LV) hypertrophy occurs secondary to an increase in myocardial cellular mass rather than ECV and that changes become increasingly prominent with increasing aerobic capacity (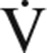
O_2max_).

## Methods

Male athletic participants aged between 20 and 45 years were approached via athletic societies and associations. Recruitment was open to athletes competing at regional, national, and international level. Subjects were eligible for inclusion if they took part in regular competition and trained for a minimum of 6 hours per week.

In addition, untrained males who exercised <3 hours per week were recruited from the study institution as a control group. Untrained individuals did not undertake regular athletic training. Subjects were not eligible if they had contraindications to CMR, hypertension (systolic blood pressure >140 mm Hg), or systemic medical illness. All athletes underwent a CMR study before a maximal exercise test, performed on the same day when logistically possible.

The study was conducted in accordance with the Declaration of Helsinki and was approved by the local ethics committee (Research and Ethics Committee reference: 14/YH/0126). All subjects gave informed written consent.

### Cardiac Magnetic Resonance Protocol

The CMR study was performed at rest and before exercise testing in athletes. All studies were performed on a 3-Tesla Achieva TX system equipped with a 32-channel cardiac phased array receiver coil and multitransmit technology (Philips Healthcare, Best, The Netherlands). The cardiac long and short axes were determined using standard scout views. Mid-LV native (precontrast) T1 maps were generated using a previously described MOLLI sequence^7^ planned using the 3 of 5 method^8^ briefly comprising: ECG triggered 5b(3s)3b MOLLI, flip angle 35°, voxel size of 1.98×1.98×10 mm^3^. LV mass and volumes were obtained from cine imaging covering the entire LV in the short axis: balanced SSFP, voxel size 1.2×1.2×10 mm^3^, no gap, 50 cardiac phases. Right ventricular and atrial volumes were obtained from a transaxial stack covering the entire heart: balanced SSFP, voxel size 1.7×1.5×5 mm^3^, no interslice gap. 0.15 mmol/kg Gadovist (Bayer Schering) was delivered by power injector (Medrad Inc, Warrendale, PA) as a single bolus via a venous cannula placed in the antecubital fossa, followed by a 20 mL saline flush at 5 mL/s. Late gadolinium enhancement (LGE) imaging (inversion recovery-prepared T1-weighted gradient echo, inversion time according to Look-Locker scout, TR/TE/flip angle 3.7 ms/2.0 ms/25°, acquired spatial resolution 1.54×1.75×10 mm) was performed to image the entire LV 7 to 10 minutes after contrast administration. Post-contrast T1 maps were acquired using the same MOLLI scheme 10 minutes after contrast administration.

### Image Analysis

All image analysis was performed using cmr42 software (Circle Cardiovascular Imaging Inc, Calgary, Alberta, Canada). Volumetric and mass analysis was performed in the standard manner from the short-axis stack^9^ (LV) or long-axis cine images^10^ (right ventricular, left and right atria). Ventricular and atrial measurements were indexed to body surface area. The presence of focal fibrosis or scar was assessed qualitatively from LGE imaging. T1 values were calculated from source images using manual motion correction, with a region of interest in the mid-inferoseptum as per Rogers et al.^11^ Partition coefficient (*λ*) and ECV were calculated using the formulae:


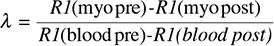



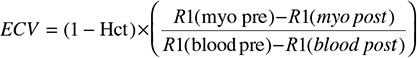


where *R*1=1/*T*1 and Hct is hematocrit.

CMR chamber and tissue values were indexed to body surface area and estimated lean body mass (height^2.7^). Indexed myocyte and extracellular mass were calculated using the formulae: indexed myocyte mass=indexed LV mass×(100−%ECV); indexed extracellular mass=indexed LV mass×%ECV. (The standard deviation of ECV measurement in our center in a cohort of 30 healthy individuals was 2.8%.) All T1, ECV, volumetric, and mass analyses were performed by 2 observers (A. K. McDiarmid and B. Erhayiem) blinded to all subject data, including sporting discipline and aerobic capacity.

### Exercise Protocol

Participants were instructed to arrive rested (no strenuous exercise in the preceding 24 hours) and having abstained from any alcohol (preceding 24 hours), food, and caffeine (preceding 3 hours) ingestion. To determine maximal oxygen uptake (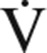
O_2max_) and ventilatory threshold, participants undertook a ramp-incremental step-exercise test on an electronically braked cycle ergometer (Excalibur Sport, Lode BV, Groningen, the Netherlands), which allows for confirmation of VO_2max_ in a single test.^12^ Participants wore a nose clip and breathed through a low-dead space, low-resistance mouthpiece which was connected to a bidirectional pitot tube flow sensor and gas sample line assembly, allowing for breath-by-breath measurement of gas volumes and concentrations (O_2_, Galvanic; CO_2_ infrared) and subsequent calculation of ventilatory and pulmonary gas exchange variables (Cardio2, Medical Graphics Corporation, St Paul, MN). Before each test, the pitot tube flow sensor was calibrated over a range of flow rates using a 3 L syringe, whereas the gas analysers were calibrated using precision gases that spanned the inspired and expired physiological range. A 12-lead ECG was monitored throughout, and heart rate was measured from the R-R interval. The ramp-incremental step-exercise test was preceded by a rest period (≈2 minutes) and unloaded cycling (20 W; ≈4 minutes) and continued until a steady state was attained, after which work rate increased as a linear function of time at a rate of 20 to 30 W/min (depending on reported training history), with the intention of bringing participants to the limit of tolerance in ≈10 to 12 minutes.^13^ The ramp increment was then followed by 5 minutes of active recovery (20 W), after which a step exercise (SE) was performed at 95% of the RI work rate peak, with this SE also continued to the limit of tolerance. In both RI and SE parts of the test, the limit of tolerance was defined as the point at which cycling cadence fell below 50 rpm, despite strong verbal encouragement.

Breath-by-breath data were edited using the 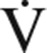
O_2_ response to eliminate erroneous breaths (occurring outside the local mean 99% prediction limits) that were considered unphysiological.^14^ Anaerobic threshold was then estimated using the V-slope method^15^ and supporting ventilatory and pulmonary gas exchange criteria (ie, the fractional end-tidal concentrations of O_2_ and CO_2_, and the ventilatory equivalents for O_2_ and CO_2_^16^). 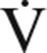
O_2peak_ was identified in both RI and SE phases as the highest 12-breath rolling average (highest mean 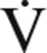
O_2_ over ≈15–20 s).^12^ Within subjects, the highest 12-breath rolling average from RI and SE phases were then compared using unpaired *t* tests, with no difference (*P*>0.05) between RI and SE 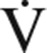
O_2peak_, and thus the attainment of 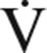
O_2max_ was confirmed in each test.^17^

### Statistical Analysis

Statistical analysis was performed using IBM SPSS Statistics 20.0 (IBM Corp, Armonk, NY). Subjects were grouped as follows: untrained, athletes with 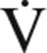
O_2max_ <60 mL/kg per min (low-performance group), and athletes with 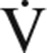
O_2max_ >60 mL/kg per min (high-performance group). Unless otherwise stated, the results are presented as mean±standard deviation (SD). Normality of distribution was determined with Kolmogarov–Smirnov testing. Differences between groups were assessed using the Chi-squared test, independent *t* test, or 1-way analysis of variance when appropriate. Post hoc analysis was performed with Bonferroni testing. Correlation was assessed with Spearman correlation coefficient. Significance for all tests was assumed with *P*<0.05.

## Results

### Study Participant Demographics and Characteristics

Thirty endurance athletes (7 runners, 11 cyclists, and 12 triathletes) and 15 untrained sedentary controls were recruited. Athletic and untrained participants were prospectively matched for age (31.7±7.7 versus 30.3±8.1 years; *P*=0.54). Hematocrit was not significantly different between groups (0.46±0.3 versus 0.46±0.3 g/dL; *P*=0.99), but body mass index was significantly lower in athletes (22.8±1.9 versus 24.4±2.5 kg/m^2^; *P*=0.02). CMR study and maximal exercise testing were separated by a median of 0 days (quartile range 1–3). Full study participant information may be seen in Table 1.

**Table 1.**
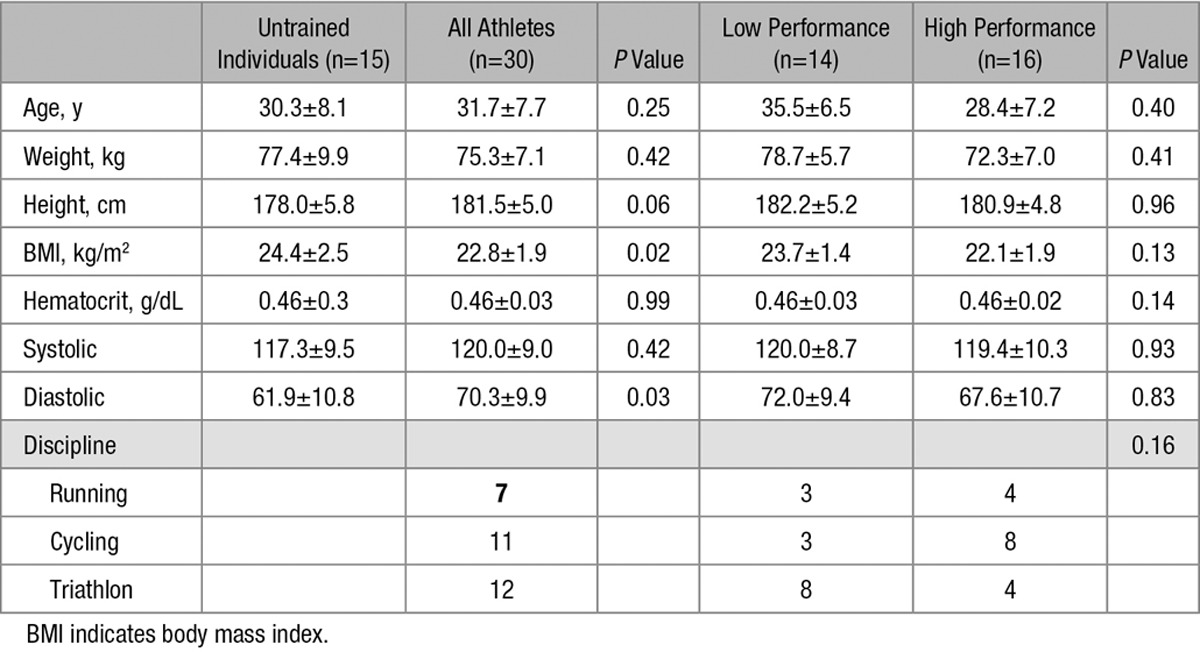
Subject Characteristics

### Performance Stratification and Cardiopulmonary Exercise Testing

Athletes were divided into high- and low-performance groups at a threshold 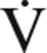
O_2max_ of 60 mL/kg per min (n=16 and 14, respectively). Mean 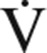
O_2max_ and anaerobic threshold as a percentage of 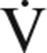
O_2max_ in high-performance and low-performance athletes, respectively, were 65.5±6.2 versus 54.2±4.5 mL/kg per min (*P*<0.001) and 61.9±6.5% versus 59.5±7.6% (*P*=0.36). No significant atrial or ventricular ectopy was recorded in the exercise or recovery phase of the exercise protocol. None of resting, peak, or Δheart rate differed between groups. Maximal exercise test results are summarized in Table 2.

**Table 2.**
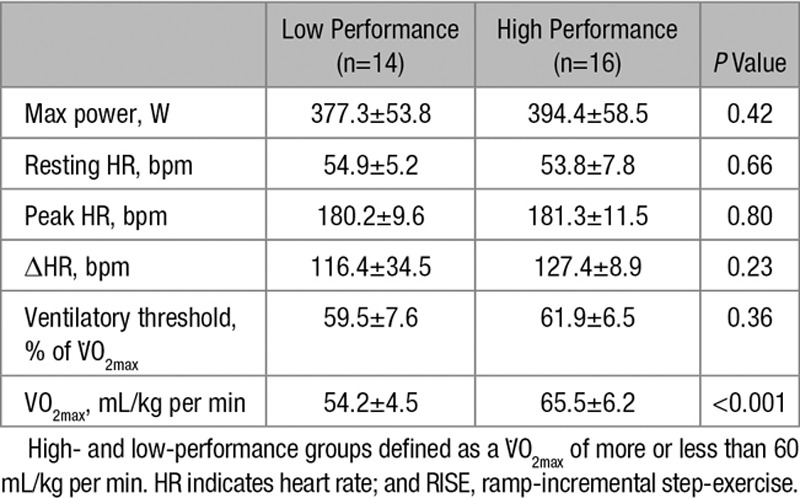
RISE Maximal Exercise Test Results

### Myocardial Remodeling

**Figure 1.**
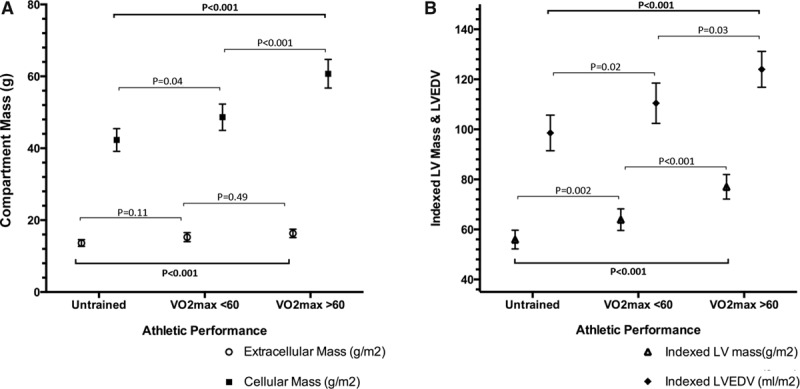
Myocardial remodeling by performance group. **A**, Cellular mass increases out of proportion to increase in extracellular mass. **B**, Indexed left ventricular end-diastolic volume (LVEDV) and left ventricular (LV) mass increases stepwise as athletic grouping changes (*P*<0.001 for both).

Indexed cellular mass was greater in high-performance athletes than in low-performance athletes (60.7±7.5 versus 48.6±6.3 g/m^2^; *P*<0.001); however, extracellular mass remained constant (16.3±2.2 versus 15.3±2.2 g/m^2^; *P*=0.20; Figure 1). Myocardial ECV and native T1 in athletes were lower than in untrained controls (22.5±2.6% versus 24.5±2.2%, *P*=0.02; 1178±32 versus 1202±33 ms, *P*=0.02). Although both the cellular and extracellular masses were higher in athletes than in controls (15.8±2.2 versus 13.6±1.7, *P*<0.001; 55.1±9.2 versus 42.3±5.7, *P*<0.001), the relative expansion of the cellular compartment was greater than the increase seen of the extracellular compartment (130% and 116%, respectively).

### Chamber Remodeling in High-Performance and Low-Performance Athletes

**Figure 2.**
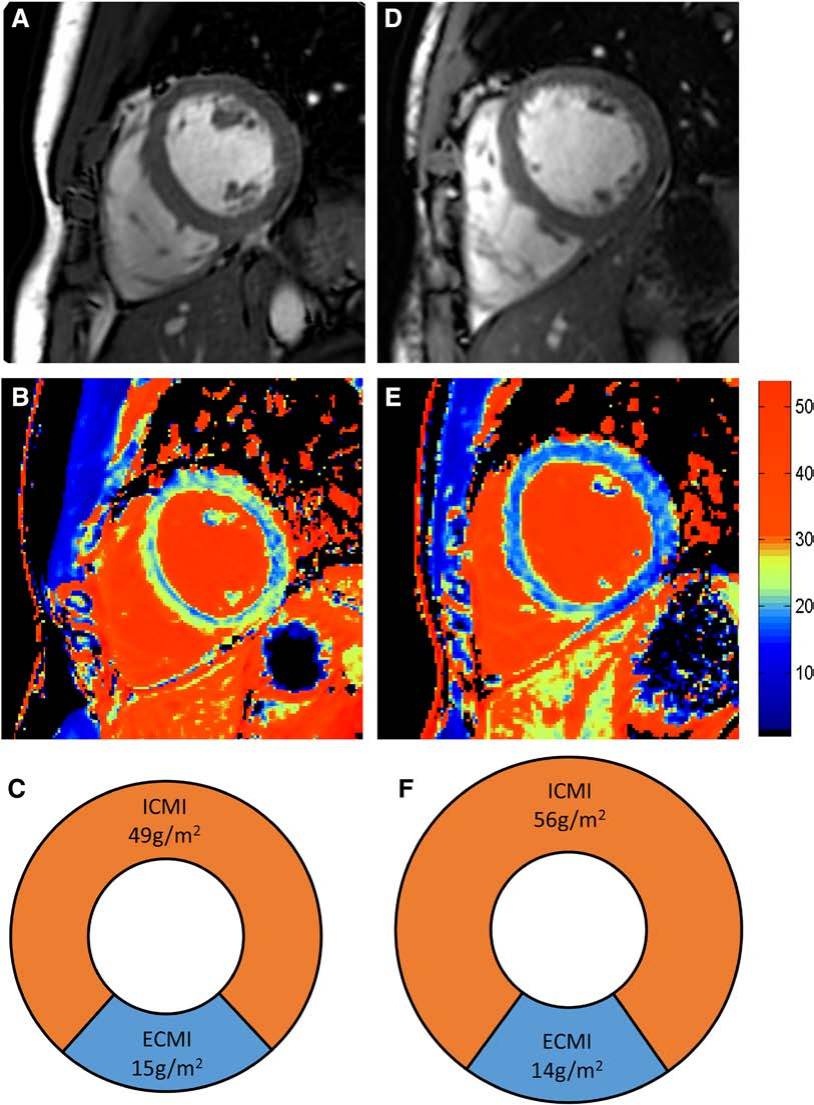
Typical cardiac magnetic resonance (CMR) appearances in low-performance and high-performance athletes. Left column shows an athlete with a 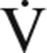
O_2max_ of 50 mL/kg per in, and right column shows a high-performance athlete with a 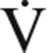
O_2max_ of 75 mL/kg per min. **A** and **D**, Short-axis images of left and right ventricle. **B** and **E**, extracellular volume (ECV) color maps showing lower ECV in the high performance athletes throughout the myocardium. **C** and **F**, Pie charts scaled to overall indexed LV mass displaying the relative indexed masses of the cellular and extracellular compartments (not short axis images). ECMI indicates indexed extracellular mass; and ICMI, indexed intracellular mass.

**Figure 3.**
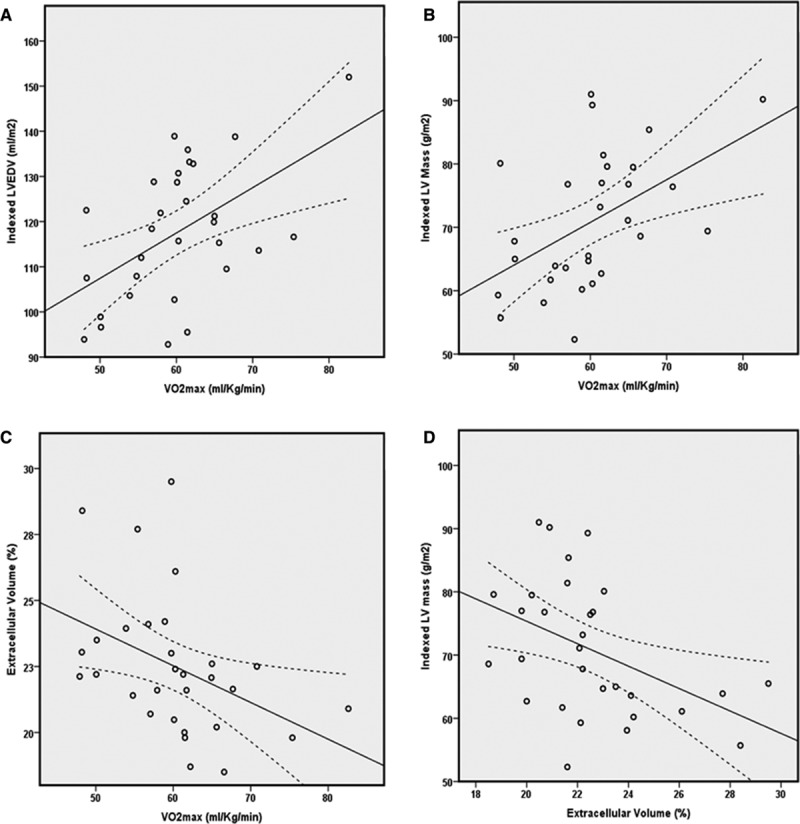
Relationship between exercise capacity and left ventricular (LV) remodeling and remodeling and tissue composition (R and P values determined with Spearman correlation coefficient for all). **A**, LVEDVi and VO_2max_, *r*=0.45, *P*=0.01; **B**, LVMi and VO_2max_, *r*=0.55, *P*=0.002; **C**, extracellular volume and VO_2max_, *r*=−0.53, *P*=0.003; **D**, significant inverse relationship between extracellular volume (ECV) and indexed LV mass (*r*=−0.44, *P*=0.02). LVEDVi indicates LV end-diastolic volume index; and LVMi, left ventricular mass index.

Typical findings may be seen in Figure 2, and full CMR data are given in Table 3. Both left ventricular mass index and left ventricular end-diastolic volume index differed significantly between athletes and untrained controls and between athletic groups (*P*<0.001 for all; Figure 2). Significant correlations were observed between cardiac chamber remodeling and 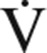
O_2max_: both left ventricular mass index and left ventricular end-diastolic volume index correlated significantly with each other (*r*=0.71, *P*<0.001) and with 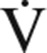
O_2max_ (*r*=0.55, *P*=0.002 and *r*=0.45, *P*=0.01, respectively; Figure 3). In athletes, CMR measures of tissue composition (ECV and partition coefficient) differed between athletic groups and correlated significantly with 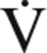
O_2max_: ECV *r*=−0.53, *P*=0.003; partition coefficient *r*=−0.41, *P*=0.02. Native T1 did not differ between athletic groups (low versus high performance; 1185±36 versus 1171±28 ms; *P*=0.26). LGE was seen in one athlete only (3%) and was in a typical myocarditis pattern. A significant inverse correlation was seen between ECV and left ventricular mass index (*r*=−0.44, *P*=0.02; Figure 3).

**Table 3.**
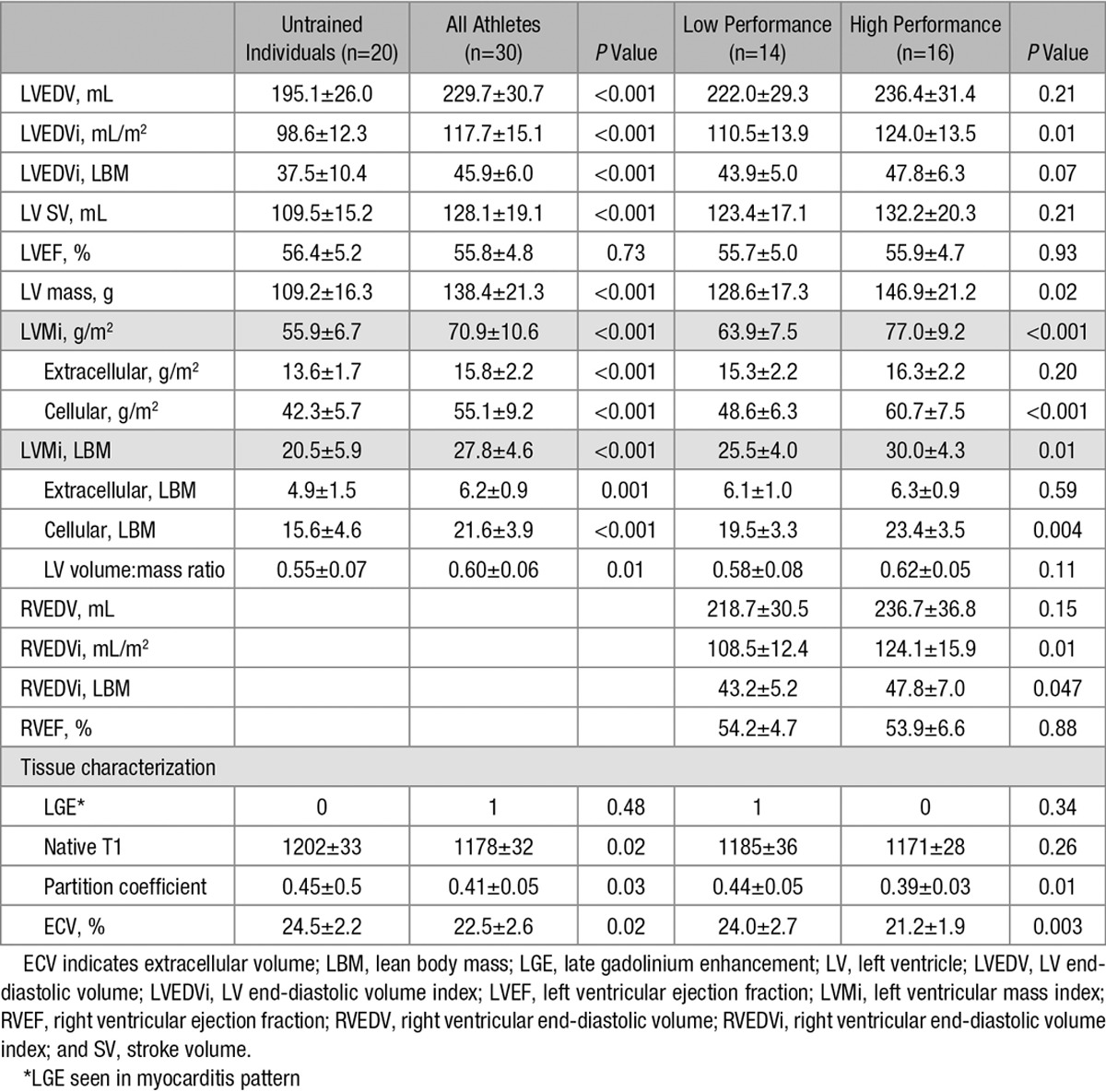
Cardiac Magnetic Resonance Findings

## Discussion

This study shows for the first time that LV hypertrophy in AH occurs as a consequence of differences in the relative composition of myocardium and that athletic hypertrophy is driven by an expansion of the cellular compartment with a relative decrease of the extracellular compartment. Furthermore, we have provided evidence of a potential relationship between increasing fitness and expansion of the cellular compartment. This finding provides novel insight into the physiological change underpinning the poorly understood phenomenon of athletic LVH. We have further demonstrated that the extent of cellular volume expansion is linearly related to aerobic capacity in this cohort of endurance athletes.

### Mechanism of Myocardial Adaption and Remodeling

Cardiovascular adaptation occurs in response to hemodynamic challenge and prolonged endurance training and even in previously untrained individuals leads to marked changes in cardiac geometry.^18^ It has been shown in previous longitudinal studies that early adaption is characterized by an increase in LV mass and as a consequence change in LV mass:volume ratio, later followed by LV dilation, normalization of the ratio, and eccentric hypertrophy.^18^ LV geometry and particularly LV mass have previously been shown to be related to 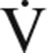
O_2max_.^19–21^

CMR has provided new insights into the mechanisms that underpin cardiac remodeling with exercise training. The multiparametric assessment of the human heart and high reproducibility provided by the technique allow both accurate functional and anatomic assessment.^22–24^ Native T1 and ECV measurement are robust and validated techniques for tissue characterization,^25^ and the correlation of ECV in particular with histological tissue specimens is excellent,^22,23,26^ though has not previously been applied to athletes with known 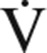
O_2max_. ECV increases in myocardial fibrosis, edema, and expansion of the extracellular space, with subsequent relative decrease in myocyte mass.^27^ Conversely, an expanded cellular mass reduces ECV as the distribution volume for conventional extracellular contrast agents is reduced.

In this study, we have demonstrated that indexed LV mass is correlated with aerobic capacity and that indexed LV mass and ECV are inversely related. Participants with a higher 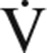
O_2max_ had a similar extracellular mass as those with a lower 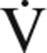
O_2max_, but a significantly higher intracellular mass.

These data allow postulation of the mechanism underlying the development of AH that is consistent with known concepts: After rapid division in fetal life, cardiac myocytes are terminally differentiated shortly after delivery. As a result, any increase in overall myocardial mass is secondary to myocyte hypertrophy or extracellular matrix expansion rather than cell hyperplasia.^28^ CMR allows in vivo quantification of the 2 tissue compartments, and our data show that in AH, the overall extracellular compartment volume is similar to previously reported normal ranges,^29^ although there is marked cellular expansion.

Important differential diagnoses of AH include dilated cardiomyopathy (DCM) and hypertrophic cardiomyopathy (HCM), which may both display increased LV mass or left ventricular end-diastolic volume. The role of CMR in the detection of these myocardial diseases is established, and both DCM and HCM display characteristic morphological abnormalities on CMR. At a microscopic level, HCM is characterized by myocyte disarray and interstitial fibrosis^30^ and, thus, higher ECV. LGE CMR allows detection of focal replacement fibrosis and infarction in both HCM^31^ and DCM^32^; however, diffuse processes are poorly detected with this technique. T1 mapping and ECV measurement have shown expansion of the extracellular space in HCM, occurring in both hypertrophic^23,29^ and nonhypertrophic segments, as well as in DCM.^33,34^ Our observation that hypertrophy in AH is cellular without, or with relatively less, ECV expansion suggests that T1 mapping CMR may be used to distinguish between athletic LV adaptation and pathological hypertrophy or remodeling.

Both side-by-side sarcomere addition in concentric remodeling and end-to-end addition in eccentric remodeling result in myocardial hypertrophy,^35^ with an increase of muscle mass without increase in myocyte number.^36^ In this study, conducted without tissue biopsy, we are unable to determine the intracellular sarcomere arrangement; however, other CMR techniques that allow myocyte size to be quantified, as investigated by Coelho-Filho et al,^37^ may allow this to be determined in the future.

### LGE and Myocardial Scar

LGE has previously been demonstrated in ≤13% of elite^38^ and 50% of veteran athletes.^39^ In this cohort of athletes, LGE was seen in only 3% (n=1). This difference may be as a consequence of the relatively young age of athletes studied.

### Remodeling and Relationship With Performance

The relationship between aerobic capacity and LV remodeling is known.^19,20^ In this study, we have demonstrated that left ventricular end-diastolic volume index and left ventricular mass index correlate with 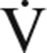
O_2max_, confirming that the degree of remodeling is related to fitness, and our data are consistent with the literature.

### Limitations

There was relatively little diversity in the range of athletic discipline pursued; however, this has previously been shown not to affect the phenotype of cardiac remodeling.^40^ Native T1 differed significantly between athletes and controls, but not athletic groups. Native T1 is increased in myocardial edema and inflammation, but also in increased extracelluar volume; consequently, these findings are consistent with ECV in this study. The reason for a lack of difference between athletic groups is not clear, however, and merits further study. Furthermore, the assumptions made regarding the mode of myocardial hypertrophy in AH in this study have not been validated by histological sample. We have not studied female athletes in this study in order to have a homogenous study sample, but it is possible that athletic adaption differs compared with men. Finally, the participants were all of white origin, and this study should be repeated in a range of ethnic origins in an effort to understand AH in all athletes because risk and remodeling may differ between ethnicities.^41^

### Conclusions

Cardiac remodeling and LVH in AH occurs as a consequence of greater myocyte mass, with an associated relative decrease of the extracellular space. This is unlike HCM or DCM, where CMR tissue characterization detects marked expansion of the extracellular space, in the presence or absence of LVH. Athletic remodeling, both on a macroscopic and microscopic level, is associated with the degree of aerobic capacity. ECV quantification with CMR may have a future role in differentiating AH from change secondary to cardiomyopathy, especially HCM, and warrants further study.

## Acknowledgments

We are grateful for the support and assistance of the radiographers (Gavin Bainbridge, Margaret Saysell, Caroline Richmond, and Stephen Mhiribidi) assistants (Deborah Scarlett and Ann Heald) during this project.

## Sources of Funding

Dr McDiarmid is funded by a British Heart Foundation (BHF) Project Grant (PG/14/10/30641). Dr Swoboda is funded by BHF Clinical Fellowship (FS/12/88/29474). Dr Plein is funded by BHF Senior Research Fellowship (FS/10/62/28409).

## Disclosures

Drs Greenwood and Plein received a research grant from Philips Healthcare. The other authors report no conflicts.

## CLINICAL PERSPECTIVE

The need for reliable methods to differentiate physiological and pathological left ventricular hypertrophy are brought into focus by the rare but prominent cases of sudden death in elite athletes and the young. However, the myocardial changes that occur in an athlete’s heart are poorly understood, and current assessment is dependent on a constellation of findings, but may be limited when significant abnormalities are not present. Consequently, differentiating between physiological and pathological left ventricular hypertrophy can be difficult in borderline cases. Cardiac magnetic resonance provides highly reproducible assessment of cardiac structure and function. The developing field of cardiac magnetic resonance T1 mapping is now allowing insights into tissue composition with quantification of the extracellular space, not previously possible with contrast-enhanced cardiac magnetic resonance. In this study, we have used T1 mapping to demonstrate that increased left ventricular mass in physiological hypertrophy is predominantly because of expansion of the cellular, rather than the extracellular compartment. Furthermore, the degree of myocardial adaptation and cellular expansion is related to aerobic capacity, with fitter individuals displaying more expansion of the cellular compartment. Relatively lower extracellular volume is a crucial difference from many cardiomyopathies that are characterized by myocardial fibrosis and expansion of the extracellular space. The relatively lower extracellular volume in physiological left ventricular hypertrophy may provide a foundation for future studies to test whether extracellular volume quantification can differentiate the underlying nature of left ventricular hypertrophy.
